# Lipid Parameters and Their Association With Liver Function Test Variables in Patients With Type 2 Diabetes Mellitus

**DOI:** 10.7759/cureus.92671

**Published:** 2025-09-18

**Authors:** Sai Venkatram Reddy Molugu, Chaitra Reddy Molugu, Hemanth Reddy Molugu, Srinivasa Reddy Badvel

**Affiliations:** 1 Medicine, Osmania Medical College, Hyderabad, IND; 2 Medicine, Kamineni Academy of Medical Sciences and Research Centre, Hyderabad, IND; 3 Medicine, Apollo Institute of Medical Sciences and Research, Hyderabad, IND; 4 Department of General Medicine, Nimra Institute of Medical Sciences, Vijayawada, IND

**Keywords:** cardiac function, dyslipidemia, fasting blood sugar, non-alcoholic fatty liver disease, type 2 diabetes

## Abstract

Aim

To assess the prevalence of elevated liver enzymes and their correlation with lipid profile abnormalities in patients with type 2 diabetes mellitus (T2DM).

Method

A total of 130 individuals with diabetes were enrolled in the study, and blood serum samples were collected to evaluate liver function tests and lipid profiles. The association between liver enzyme levels and T2DM was analyzed using logistic regression.

Results

Altered liver enzymes were observed in 40 cases (30.77%), while lipid abnormalities were noted in 92 cases (70.76%) in T2DM cases. Among liver function parameters, aspartate aminotransferase (AST) and alanine aminotransferase (ALT) exhibited the greatest degree of variation. AST showed significant correlations with total cholesterol (r = 0.513), triglycerides (r = 0.530), low-density lipoprotein (LDL) (r = 0.518), diabetes duration (r = 0.198), and high-density lipoprotein (HDL) (r = -0.288). Similarly, ALT showed significant correlations with all lipid profile parameters. Logistic regression analysis revealed that abnormal levels of total cholesterol (OR: 1.452, p < 0.0001; 95% CI: 1.29-1.62), LDL (OR: 1.59, p < 0.0001; 95% CI: 0.41-0.69), and triglycerides (OR: 1.798, p < 0.0001; 95% CI: 1.699-2.12) were independently associated with type 2 diabetes mellitus. Receiver operating characteristic (ROC) curve analysis showed that ALT had an AUROC of 0.86 in predicting non-alcoholic fatty liver disease (NAFLD) among diabetic patients. There was a strong correlation between abnormal liver enzymes, and dyslipidemia, with odds ratio analysis indicating an increased risk of liver disorders in individuals with poor diabetic control. Liver function abnormalities frequently coexist with altered lipid profiles in patients with T2DM.

Conclusion

Consistent monitoring of lipid profile levels in patients with T2DM may aid in preventing cardiovascular complications. Follow-up using laboratory parameters such as Fibrosis-4 (FIB-4) scores and elastography is recommended to monitor patients with fatty liver disease, as these variables can detect early hepatic changes, advanced fibrosis, and other liver abnormalities. Regular screening for metabolic dysfunction-associated steatotic liver disease (MASLD) may be warranted in diabetic patients with elevated liver enzymes, potentially facilitating early intervention and reducing the risk of cirrhosis and its related complications.

## Introduction

According to the International Diabetes Federation Diabetes Atlas, in 2021, approximately 10.5% of the global population aged 20 to 79 was affected by diabetes, equating to around 536.6 million people. Projections indicate that this prevalence is expected to increase to 12.2%, or nearly 783.2 million individuals, by 2045 [1,2]. A study by the Indian Council of Medical Research-India Diabetes reveals that India has 62.4 million confirmed cases of type 2 diabetes mellitus (T2DM), in addition to 77 million people classified as pre-diabetic [3,4]. Globally, more than 415 million people aged 20 to 79 are currently diagnosed with diabetes mellitus, with forecasts suggesting this figure could rise to 642 million by 2040 [5]. Studies indicate that individuals with T2DM exhibit a higher prevalence of liver function test abnormalities compared to those without diabetes. These findings suggest that elevated liver enzyme levels may serve as indicators of reduced insulin sensitivity, increased insulin resistance, and the development of T2DM [6].

Patients with T2DM often display an abnormal lipid profile, which includes high triglyceride (TG) levels, lipoproteins rich in fat, decreased high-density lipoprotein (HDL) cholesterol, and an increased level of low-density lipoprotein (LDL). These lipid abnormalities not only pose significant health threats but also serve as a major risk factor for cardiovascular diseases, which are well-established and present serious challenges for those with diabetes [7-9]. The onset of T2DM may be linked to increased lipolysis and the release of free fatty acids from fat cells due to insulin resistance, which in turn stimulates heightened lipid production in liver cells, contributing to the dyslipidemia seen in T2DM. Previous research has shown an association between alanine aminotransferase (ALT) activity, dyslipidemia, and insulin resistance in T2DM patients [8-10].

Evidence shows a significant, bidirectional relationship between liver function and diabetes, where liver dysfunction can cause or worsen diabetes, and diabetes can, in turn, damage the liver [11]. While insulin resistance is a central culprit, the full mechanism is complex and involves several interconnected biological pathways. But, its mechanism needs to be fully understood.

This study aims to investigate the association between lipid profiles and liver function tests (LFTs) in individuals with T2DM. The main goal is to facilitate the early identification of metabolic dysfunction-associated steatotic liver disease (MASLD) and to minimize the risk of its progression to non-alcoholic steatohepatitis (NASH) or cirrhosis.

Primary objective is to assess the prevalence of liver function test abnormalities and their associated factors among patients with T2DM attending the hospital.

## Materials and methods

This cross-sectional study was conducted at the General Medicine department for the duration of 18 months. Sample size calculation was done based on a previous study [12]. Based on the formula n = (Z^(2 α/2) p(1-p))/E^2, the calculated sample size was 128.21, where n = required minimum sample size, p = prevalence of diabetes mellitus (9.3%), α = level of significance, Zα = the z score corresponding to the degree of confidence, and E = desired precision/absolute precision is 5%. 

The study included patients diagnosed with T2DM who were visiting the General Medicine Outpatient Department. The study protocol received approval from the Institutional Ethics Committee at NIMS, Vijayawada (IEC/NIMS-VJA/2023-GM.03; dated 14-04-2023). Written informed consent was obtained from all participants prior to inclusion in the study. Patients with chronic alcoholism, alcoholic fatty liver, liver cirrhosis, liver malignancy, or those suffering from drug or viral hepatitis were excluded. A variety of tests were performed, including a complete blood count, liver function tests, fasting lipid profile, fasting blood sugar, postprandial blood sugars, and abdominal ultrasound. The diagnostic criteria for type 2 diabetes mellitus adhered to the 2023 guidelines established by the American Diabetes Association (ADA), which defined diabetes as a fasting blood sugar level of 126 mg/dl or higher, a 2-hour oral glucose tolerance test result of 200 mg/dl or more, and glycated hemoglobin (HbA1c) of 6.5% or greater.

The study involved a group of 130 individuals diagnosed with T2DM who were receiving treatment at the General Medicine outpatient department. Participants underwent a thorough evaluation, which included fasting blood sugar, postprandial blood sugar, liver function tests, fasting lipid profiles, and abdominal ultrasounds to assess for fatty liver. Those with an ALT/aspartate aminotransferase (AST) ratio exceeding one, total cholesterol levels above 200 mg/dl, and TG levels greater than 150 mg/dl were identified as having elevated levels, and the relationship between these factors and fatty liver was investigated (Table 1).

**Table 1:** Laboratory variables reference ranges HbA1c: glycated hemoglobin, HDL: high density lipoprotein, mg/dl: milligram/deciliter

Variables	Reference ranges
Blood glucose	
Fasting blood glucose	60-100 mg/dl
Post-prandial blood glucose	80-140 mg/dl
Random blood glucose	60-110/dl
HbA1c	4.0-6.0%
serum triglycerides	
Normal	< 150 mg/dl
High	200-400mg/dl
Elevated	>400 mg/dl
serum HDL cholesterol	
Low	< 40 mg/dl
Desirable	40-60 mg/dl
serum total cholesterol	
Desirable	< 200 mg/dl
Borderline	200 - 240 mg/dl
High	> 240 mg/dl

Ultrasound features

Liver parenchyma appears more echogenic (“brighter”) than the renal cortex or spleen in patients with increased echogenicity, such as poor visualization or obscuration of intrahepatic vessels (portal and hepatic veins) in patients with vascular blurring. Posterior portions of the liver appear less distinct due to attenuation of the beam by fatty infiltration in patients with deep attenuation of ultrasound beam. The diaphragm appears poorly visualized or blurred in patients with loss of diaphragmatic clarity. Grading of fatty liver is distributed from mild to severe. Mild shows diffuse increase in liver echogenicity with normal visualization of diaphragm and intrahepatic vessel borders in patients with grade 1. Moderate shows diffuse increase in echogenicity with slightly impaired visualization of intrahepatic vessels and diaphragm in patients with grade 2. There is a marked increase in echogenicity with poor or absent visualization of intrahepatic vessels, diaphragm, and posterior liver segment in grade 3 fatty liver. 

The HumaLyzer-3500 automated chemistry analyzer with corresponding kits (Human Diagnostics, Wiesbaden, Germany) was used for all biochemical assessments. Serum tests included fasting glucose, postprandial glucose, total cholesterol (TC), TG, HDL, ALT, AST, and gamma-glutamyl transferase (GGT).

Statistical analysis

Data collected from participants were recorded in an Excel spreadsheet (Microsoft, Redmond, WA, USA) and imported into SPSS version 24.0 (IBM Corp., Armonk, NY, USA) for statistical analysis. To investigate the relationships between dependent and independent variables, binary logistic regression and bivariate correlation analyses were conducted. The Student's t-test was used to compare two unpaired groups, with a significance level set at 0.0001. Pearson’s correlation analysis was performed to assess correlations, with correlation coefficients (r value) reported at a significance level of 0.05. The Chi-square and Mann-Whitney tests were utilized to compare qualitative and quantitative data, with p-values considered significant at 0.05 and highly significant at 0.001. Qualitative categorical variables were expressed as counts and percentages.

## Results

Among the total of 130 patients with T2DM, 67 were males and 63 were females. The mean age was 54.13±8.85 years (range: 35-77 years). Out of 130 participants, 90 (69.23%) had normal LFT values, while 40 patients (30.77%) demonstrated abnormalities in at least one LFT parameter. Lipid abnormalities were identified in 92 patients (70.76%). Specifically, total cholesterol levels exceeded 200 mg/dL in 47 participants (36.15%), elevated LDL levels (>100 mg/dL) were found in 92 individuals (70.76%), TG levels surpassed 150 mg/dL in 82 cases (63.07%), and low HDL levels (<40 mg/dL) were observed in 50 subjects (38.46%). According to the Centers for Disease Control and Prevention (CDC), approximately 97% of adults with diabetes present with one or more lipid abnormalities, while reported prevalence of diabetic dyslipidemia across studies ranges between 25% and 60%, likely due to variations in body mass index (BMI) and genetic factors. The frequency of altered liver enzymes in the study population was 40 patients (30.77%), including 22 males and 18 females. The mean fasting blood sugar (FBS) level in patients with normal liver function was 167.74±23.43 mg/dL, compared to 194.9±24.78 mg/dL in those with abnormal liver function; however, this difference did not reach statistical significance (Table 2).

**Table 2:** Distribution of abnormal liver function in patients with type 2 diabetes mellitus data is represented as N, %, LFT: liver function test, AST: aspartate aminotransferase, ALT: alanine aminotransferase, ALP: alkaline phosphatase, GGT: gamma-glutamyl transferase, g/dL: grams/deciliter, mg/dL: milligrams/decilitres, U/L: units/liter

Parameters	Normal range	Normal LFT	abnormal LFT	% of Abnormality
Total bilirubin (mg/dL)	0.3-1.2 mg/dL	91	39	30%
AST (U/L)	8-48 U/L males: 8-48 U/L females: 8-43 U/L	63	67	51.54%
ALT (U/L)	7-55 U/L males: 7-56 U/L females: 7-45 U/L	90	40	30.76%
ALP (U/L)	44-147 U/L	56	74	56.93%
Total protein (g/dL)	6.0-8.3 g/dL	89	41	31.54%
Albumin (g/dL)	3.4-5.4 g/dL	102	28	21.54%
GGT	5-40 U/L	90	40	30.76%

The mean values of TC, TG, and LDL with normal liver function were observed to be 180.3667±27.767 mg/dL, 171.089±95.58mg/dL, and 114.655±25.687 mg/dL, respectively, while with abnormal liver function, they were observed to be 224.575±49.927 mg/dL, 340.575±317.26 mg/dL, and 154.075±45.0158 mg/dL, respectively with statistical significant difference (p<0.0001) (Table 3).

**Table 3:** Comparison of demographics and biochemical parameters between normal and abnormal liver function test variables data is represented as mean±standard deviation, LFT: liver function test, BMI: body mass index, LDL: low-density lipoprotein, HDL: high-density lipoprotein, VLDL: very low-density lipoprotein, mg/dL: milligrams/deciliters, kg/m2: kilograms/metres square, p-value is considered significant at p<0.05

Parameters	Mean±SD		p- value
	Normal LFT	Abnormal LFT	
Age (years)	53.8889±9.03	54.675±8.48	0.26
BMI (kg/m2)	24.9222±3.01	26.325±4.12	0.29
Fasting glucose (mg/dL)	167.744±23.43	194.9±24.781	0.59
Total cholesterol (mg/dL)	180.3667±27.76	224.575±49.92	<0.001
Triglyceride (mg/dL)	171.089±95.58	340.575±317.26	<0.001
LDL (mg/dL)	114.655±25.68	154.075±45.01	<0.001
HDL (mg/dL)	42.3333±6.43	36.775±5.75	0.04
VLDL	27.222±9.9896	37.525±7.749	0.002

Out of 130 patients with T2DM, 47 (36.15%) had elevated TC, 92 (70.76%) had raised LDL, 82 (63.07%) had elevated TG, and 50 (38.46%) had reduced HDL levels. Lipid abnormalities were therefore observed in 92 patients (70.76%), with high LDL and low HDL being the most frequent patterns (Table 4). With respect to liver function parameters, 39 (30%) patients had elevated total bilirubin, 67 (51.5%) had raised AST, 40 (30.77%) had elevated ALT, and 74 (56.9%) had increased alkaline phosphatase (ALP). Reduced total protein and albumin levels were noted in 41 (31.5%) and 28 (21.5%) cases, respectively. The mean values of AST, ALT and total protein with normal liver function were observed to be 28.238±2.11533 U/L, 21.611±4.8452 U/L, and 7.5426±0.337 mg/dL, respectively, while with abnormal liver function, they were observed to be 59.089±22.8458 U/L, 107.925±38.6047 U/L, and 5.4±0.3619 mg/dL, respectively, showing statistical significance (p<0.0001). In our study, lipid abnormality was found in 92 (70.76%) subjects. TC of >200mg/dl was found in 47 (36.15%) cases, LDL of >100mg/dl was found in 92 (70.76%) cases, TG >150mg/dl was present in 82 (63.07%) cases, HDL of <40mg/dl was found in 50 (38.46%) subjects. High levels of LDL and low levels of HDL were of frequent occurrence (Table 4).

**Table 4:** Abnormal lipid profile in patients with type 2 diabetes mellitus data has been represented as N, %, mg/dL: milligrams/deciliters, LDL-C: low-density lipoprotein cholesterol, HDL-C: high-density lipoprotein cholesterol

Parameters	Normal range	Normal lipid profile	Abnormal lipid profile	% of Abnormality
Total cholesterol	<200mg/dl	83	47	36.16%
LDL-C	<100mg/dl	38	92	70.77%
Triglycerides	<150mg/dl	48	82	63.07%
HDL-C	>40mg/dl	80	50	38.47%

The frequencies of abnormal LFT values among the study population were as follows: total bilirubin 30% (n=39), AST 51.54% (n=67), ALT 30.76% (n=40), albumin 21.54% (n=28), and total protein 31.54% (n=41). The most frequent abnormality was elevated AST (67 cases), whereas hypoalbuminemia (28 cases) was the least common. 

AST showed significant positive correlations with fasting blood sugar (r=0.467, p<0.0001), HbA1C (r=0.269, p=0.002), total cholesterol (r=0.513, p<0.0001), triglycerides (r=0.530, p<0.0001), LDL (r=0.518, p<0.0001), and diabetes duration (r=0.198, p=0.002), while negatively correlating with HDL (r=-0.288, p<0.0001). 

ALT exhibited significant positive correlations with fasting blood sugar (r=0.547, p<0.0001), HbA1C (r=0.281, p=0.001), total cholesterol (r=0.575, p<0.0001), triglycerides (r=0.610, p<0.0001), LDL (r=0.577, p<0.0001), and diabetes duration (r=0.188, p=0.012). It was negatively correlated with HDL (r=-0.377, p<0.0001).

Total bilirubin also demonstrated strong correlations with fasting blood sugar (r=0.528, p<0.0001), HbA1C (r=0.250, p=0.001), total cholesterol (r=0.517, p<0.0001), triglycerides (r=0.497, p<0.0001), LDL (r=0.520, p<0.0001), diabetes duration (r=0.191, p=0.022), and a negative correlation with HDL (r=-0.408, p<0.0001).

Albumin was negatively correlated with fasting blood sugar (r=-0.354, p<0.0001), HbA1C (r=-0.174, p=0.048), total cholesterol (r=-0.412, p<0.0001), triglycerides (r=-0.348, p<0.0001), LDL (r=-0.396, p<0.0001), and diabetes duration (r=-0.130, p=0.01), but positively correlated with HDL (r=0.313, p<0.0001).

Total protein showed similar negative correlations with fasting blood sugar (r=-0.508, p<0.0001), HbA1C (r=-0.276, p=0.001), total cholesterol (r=-0.519, p<0.0001), triglycerides (r=-0.490, p<0.0001), LDL (r=-0.511, p<0.0001), and diabetes duration (r=-0.173, p=0.01), while positively correlating with HDL (r=0.404, p<0.0001).

Mean values of ALP, ALT, AST, and total bilirubin were significantly higher in T2DM patients with abnormal liver function compared to those with normal liver function. Conversely, albumin (2.99±0.18 vs. 5.01±0.69 g/dL) and total protein (5.40±0.36 vs. 7.54±0.34 g/dL) were significantly lower in patients with abnormal liver function (p<0.0001 for all comparisons).

Independent sample T-test comparing ALT (<40 IU/L vs. ≥40 IU/L) with clinical and biochemical parameters revealed significant associations with albumin, AST/ALT ratio, total bilirubin, HbA1C, triglycerides, LDL, total cholesterol, and waist circumference (Table 5).

**Table 5:** Association of anthropometric and biochemical variables and ALT levels *T-Test analysis, BMI: body mass index, WC: waist circumference, HC: hip circumference, WHR: waist-hip ratio, TC: total cholesterol, HDL: high-density lipoprotein, LDL: low-density lipoprotein, VLDL: very low-density lipoprotein, TG: triglycerides, ALT: alanine aminotransferase, AST: aspartate transaminase, ALP: alkaline phosphatase, FBS: fasting blood sugar, PPBS: postprandial blood sugar, HbA1C: glycated haemoglobin, SBP: systolic blood pressure, DBP: diastolic blood pressure, p-value is considered significant at p<0.05

	ALT Type	N	Mean	Std. Deviation	Std. Error Mean	F	*p value
Age (years)	ALT (< 40 IU/L)	90	53.89	9.032	0.952	0.036	0.850
	ALT (>= 40 IU/L)	40	54.68	8.486	1.342		
BMI (kg/m2)	ALT (< 40 IU/L)	90	24.9222	3.013	0.317	1.599	0.208
	ALT (>= 40 IU/L)	40	26.3250	4.129	0.652		
WC	ALT (< 40 IU/L)	90	97.59	9.667	1.019	4.254	0.041
	ALT (>= 40 IU/L)	40	101.23	13.831	2.187		
HC	ALT (< 40 IU/L)	90	100.57	8.116	0.855	3.857	0.052
	ALT (>= 40 IU/L)	40	103.40	12.224	1.933		
WHR	ALT (< 40 IU/L)	90	0.9682	0.0694	0.0073	0.002	0.964
	ALT (>= 40 IU/L)	40	0.9760	0.0704	0.0111		
TC	ALT (< 40 IU/L)	90	180.37	27.768	2.927	9.164	0.003
	ALT (>= 40 IU/L)	40	224.58	49.928	7.894		
HDL	ALT (< 40 IU/L)	90	42.33	6.434	0.678	.212	0.646
	ALT (>= 40 IU/L)	40	36.78	5.753	0.910		
LDL	ALT (< 40 IU/L)	90	114.66	25.687	2.708	20.887	0.000
	ALT (>= 40 IU/L)	40	154.07	45.016	7.118		
VLDL	ALT (< 40 IU/L)	90	27.22	9.990	1.053	0.519	0.472
	ALT (>= 40 IU/L)	40	37.53	7.749	1.225		
TG	ALT (< 40 IU/L)	90	171.09	95.580	10.075	31.669	0.000
	ALT (>= 40 IU/L)	40	340.58	317.26	50.16		
FBS	ALT (< 40 IU/L)	90	167.74	23.439	2.471	3.441	0.066
	ALT (>= 40 IU/L)	40	194.90	24.782	3.918		
PPBS	ALT (< 40 IU/L)	90	228.66	45.051	4.749	.440	0.508
	ALT (>= 40 IU/L)	40	274.00	40.967	6.477		
HbA1C	ALT (< 40 IU/L)	90	10.8333	2.04010	0.21505	8.409	0.004
	ALT (>= 40 IU/L)	40	11.6300	1.62563	0.25703		
SBP	ALT (< 40 IU/L)	90	136.84	14.179	1.495	.001	0.970
	ALT (>= 40 IU/L)	40	144.60	14.207	2.246		
DBP	ALT (< 40 IU/L)	90	88.07	6.874	0.725	1.213	0.273
	ALT (>= 40 IU/L)	40	92.10	5.887	0.931		
Total Bilirubin	ALT (< 40 IU/L)	90	0.4990	0.17696	0.01865	13.724	0.000
	ALT (>= 40 IU/L)	40	1.5475	0.23857	0.03772		
AST/ALT	ALT (< 40 IU/L)	90	1.5159	0.29491	0.03109	25.237	0.000
	ALT (>= 40 IU/L)	40	0.6654	0.08407	0.01329		
ALP	ALT (< 40 IU/L)	90	182.70	181.154	19.095	0.287	0.593
	ALT (>= 40 IU/L)	40	345.35	101.524	16.052		
Albumin	ALT (< 40 IU/L)	90	5.2033	0.46867	0.04940	6.416	0.013
	ALT (>= 40 IU/L)	40	3.1600	0.31929	0.05048		
Total Protein	ALT (< 40 IU/L)	90	7.5156	0.42265	0.04455	0.895	0.346
	ALT (>= 40 IU/L)	40	5.4075	0.36331	0.05744		

The most frequent LFT abnormality was AST elevation (51.54%, 95% CI: 29-64.2%), followed by ALT elevation (30.76%, 95% CI: 28.5-42.63%). The prevalence of abnormal ALP was 56.93% (95% CI: 45.5-65.2%). Correlation analysis of lipid parameters with LFT variables in patients with T2DM revealed the following trends: HDL showed a negative correlation with ALT, AST, and ALP. LDL demonstrated a positive correlation with ALT, AST, and ALP. TG was positively correlated with AST, ALT, and ALP levels (Table 6).

**Table 6:** Correlation analysis of biochemical parameters HDL: high-density lipoprotein cholesterol, LDL: low-density lipoprotein, VLDL: very low-density lipoprotein, TG: triglycerides, ALT: alanine aminotransferase, AST: aspartate transaminase, ALP: alkaline phosphatase, FBS: fasting blood sugar, PPBS: postprandial blood sugar, HbA1C: glycated haemoglobin, ALB: albumin, p-value is considered significant at p<0.05

		TC	HDL-C	LDL-C	VLDL-C	TG	FBS	PPBS	HbA1C	Total Bilirubin	AST	ALT	AST/ALT	ALP	ALB	Total Protein
HDL	Pearson Correlation	-0.214														
	p-value	0.014														
LDL	Pearson Correlation	0.919	-0.329													
	p-value	0.000	0.000													
VLDL	Pearson Correlation	0.606	-0.250	0.451												
	p-value	0.000	0.004	0.000												
TG	Pearson Correlation	0.664	-0.433	0.609	0.539											
	p-value	0.000	0.000	0.000	0.000											
FBS	Pearson Correlation	0.458	-0.380	0.410	0.359	0.609										
	p-value	0.000	0.000	0.000	0.000	0.000										
PPBS	Pearson Correlation	0.475	-0.452	0.480	0.367	0.599	0.805									
	p-value	0.000	0.000	0.000	0.000	0.000	0.000									
HbA1C	Pearson Correlation	0.149	-0.196	0.197	0.322	0.351	0.350	0.491								
	p-value	0.090	0.026	0.025	0.000	0.000	0.000	0.000								
Total Bilirubin	Pearson Correlation	0.517	-0.408	0.520	0.495	0.497	0.528	0.510	0.250							
	p-value	0.000	0.000	0.000	0.000	0.000	0.000	0.000	0.004							
AST	Pearson Correlation	0.513	-0.288	0.518	0.469	0.530	0.467	0.444	0.269	0.798						
	p-value	0.000	0.001	0.000	0.000	0.000	0.000	0.000	0.002	0.000						
ALT	Pearson Correlation	0.575	-0.377	0.577	0.522	0.610	0.547	0.508	0.281	0.906	0.892					
	p-value	0.000	0.000	0.000	0.000	0.000	0.000	0.000	0.001	0.000	0.000					
AST/ALT	Pearson Correlation	-0.412	0.313	-0.39	-0.414	-0.348	-0.372	-0.344	-0.174*	-0.791	-0.502	-0.774				
	p-value	0.000	0.000	0.000	0.000	0.000	0.000	0.000	0.048	0.000	0.000	0.000				
ALP	Pearson Correlation	0.392	-0.230	0.365	0.246	0.367	0.290	0.275	0.107	0.365	0.450	0.472	-0.320			
	p-value	0.000	0.008	0.000	0.005	0.000	0.001	0.002	0.226	0.000	0.000	0.000	0.000			
ALB	Pearson Correlation	-0.434	0.275	-0.429	-0.450	-0.318	-0.354	-0.317	-0.211*	-0.829	-0.724	-0.789	0.743	-0.347		
	p-value	0.000	0.002	0.000	0.000	0.000	0.000	0.000	0.016	0.000	0.000	0.000	0.000	0.000		
Total Protein	Pearson Correlation	-0.519	0.404	-0.511	-0.505	-0.460	-0.508	-0.475	-0.276	-0.886	-0.743	-0.861	0.802	-.405	0.834	
	p-value	0.000	0.000	0.000	0.000	0.000	0.000	0.000	0.001	0.000	0.000	0.000	0.000	0.000	0.000	

Correlation analysis between lipid profile and liver function parameters demonstrated that BMI, TC, TG, LDL, and FBS were significantly and positively correlated with elevated levels of ALP, ALT, AST, and total bilirubin (p < 0.001) (Table 7). Based on ultrasonography scans, 40 patients (30.76%) with T2DM were found to have non-alcoholic fatty liver disease (NAFLD), while 90 patients (69.23%) did not show fatty liver changes. Diabetic patients with NAFLD exhibited significantly higher ALT (107.93±38.60 U/L), AST (69.73±20.06 U/L), and total bilirubin (1.55±0.24 mg/dL), significantly lower AST:ALT ratio (0.665±0.084) when compared to diabetic patients without NAFLD. However, there was no significant difference in ALP levels between the two groups. 

**Table 7:** Correlation between lipid profile and liver function parameters in type 2 diabetes mellitus TC: total cholesterol, HDL: high-density lipoprotein cholesterol, LDL: low-density lipoprotein, TG: triglycerides, ALT: alanine aminotransferase, AST: aspartate transaminase, ALP: alkaline phosphatase, FBS: fasting blood sugar, HbA1C: glycated haemoglobin, p-value is considered significant at p<0.05

Parameters	Total bilirubin		AST		ALT		ALP		Total protein		Albumin		AST/ALT	
	r	p-value	r	p-value	r	p-value	r	p-value	r	p-value	r	p-value	r	p-value
FBS (mg/dL)	0.528	<0.0001	0.467	<0.0001	0.547	<0.0001	0.290	0.001	-0.508	<0.0001	-0.354	<0.0001	-0.372	<0.0001
HbA1C	0.250	0.004	0.269	0.002	0.281	0.001	0.107	0.226	-0.276	0.001	-0.211	0.016	-0.174	0.048
TC (mg/dL)	0.517	<0.0001	0.513	<0.0001	0.575	<0.0001	0.392	<0.0001	-0.519	<0.0001	-0.434	<0.0001	-0.412	<0.0001
TG (mg/dL)	0.497	<0.0001	0.530	<0.0001	0.610	<0.0001	0.367	<0.001	-0.460	<0.0001	-0.318	<0.0001	-0.348	<0.0001
LDL (mg/dL)	0.520	<0.0001	0.518	<0.0001	0.577	<0.0001	0.365	<0.0001	-0.511	<0.0001	-0.429	<0.0001	-0.396	<0.0001
HDL (mg/dL)	-0.408	<0.0001	-0.288	<0.0001	-0.377	<0.0001	-0.230	0.008	0.404	<0.0001	0.275	0.002	0.313	<0.0001
Diabetes duration	0.191	0.022	0.198	0.022	0.188	0.012	0.235	0.002	0.173	0.01	-0.130	0.01	-0.366	0.003

It was observed that BMI, TC, TG, LDL, and FBS were significantly and positively correlated with elevated levels of ALP, ALT, AST, and total bilirubin (p < 0.001). A multiple binary logistic regression analysis (stepwise method) was performed to determine the independent associations of liver function parameters and lipid profile abnormalities with diabetes (Table 8). The results showed that abnormal ALP (OR: 1.43, p < 0.0001, 95% CI: 1.29-1.64), abnormal AST (OR: 1.85, p < 0.0001, 95% CI: 1.69-2.11), and abnormal ALT (OR: 1.39, p < 0.0001, 95% CI: 1.22-1.46) were independently associated with diabetes. Similarly, among lipid profile parameters, logistic regression revealed independent associations for abnormal total cholesterol (OR: 1.45, p < 0.0001, 95% CI: 1.29-1.62), abnormal LDL (OR: 1.59, p < 0.0001, 95% CI: 1.41-1.69), and abnormal triglycerides (OR: 1.80, p < 0.0001, 95% CI: 1.70-2.12). Furthermore, receiver operating characteristic (ROC) curve analysis demonstrated that ALT had an area under the ROC curve (AUROC) of 0.86 for predicting NAFLD in diabetic patients, indicating good diagnostic accuracy. 

**Table 8:** Multiple logistic regression analysis TC: total cholesterol, HDL: high-density lipoprotein cholesterol, LDL: low-density lipoprotein, TG: triglycerides, ALT: alanine aminotransferase, AST: aspartate transaminase, ALP: alkaline phosphatase, OR: odds ratio

Parameters	Multiple logistic regression			
	Variable	p-value	OR	95% CI OR
ALP	Normal	Ref	1	–
	Abnormal	<0.0001	1.43	from 1.29 to 1.64
Bilirubin-total	Normal	Ref	1	–
	Abnormal	<0.0001	0.63	from 0.52 to 0.72
AST	Normal	Ref	1	–
	Abnormal	<0.0001	1.85	from 1.69 to 2.11
ALT	Normal	Ref	1	–
	Abnormal	<0.0001	1.39	from 1.22 to 1.46
TC	Normal	Ref	1	–
	Abnormal	<0.0001	1.452	from 1.29 to 1.62
LDL	Normal	Ref	1	–
	Abnormal	<0.0001	0.59	from 0.41 to 0.69
TG	Normal	Ref	1	–
	Abnormal	<0.0001	1.798	from 1.699 to 2.12
HDL	Normal	Ref	1	–
	Abnormal	<0.0001	1.42	from 1.33 to 1.85

## Discussion

Patients with T2DM often present with lipid abnormalities, and since the liver regulates glucose and lipid metabolism, a strong relationship is likely between lipid profiles and LFTs. Elevated LFTs can occur in diabetic individuals regardless of the presence of NAFLD.

In this study, 130 patients with T2DM attending the General Medicine Outpatient Department were evaluated. The mean age of participants was 54.13±8.85 years (range: 35-77 years). Abnormalities in at least one liver function parameter were observed in 40 individuals (30.77%). Prior study indicate that people aged 45-64 years in developing countries and those ≥65 years in developed nations are particularly vulnerable to diabetes [13].

Lipid abnormalities were common, affecting 92 subjects (70.76%). Specifically, total cholesterol >200 mg/dL was noted in 47 cases (36.15%), LDL >100 mg/dL in 92 cases (70.76%), triglycerides >150 mg/dL in 82 cases (63.07%), and HDL <40 mg/dL in 50 cases (38.46%). According to the CDC, up to 97% of adults with diabetes exhibit at least one lipid abnormality, with prevalence rates varying from 25% to 60% depending on BMI, genetics, and dietary factors [14]. Mechanistically, elevated TG promote cholesteryl ester transfer from HDL and LDL to VLDL, producing small, dense LDL particles that accelerate atherosclerosis. T2DM is also associated with low HDL levels, which further increases cardiovascular risk. Fagot-Campagna et al. reported that 70-97% of T2DM patients exhibit dyslipidemia, consistent with our findings [15].

The most common LFT abnormality in our study was elevated ALT, observed in 40 patients. Prior studies also suggest male sex is linked to abnormal LFTs, possibly due to estrogen-related differences in fat distribution [16-18]. AST correlated positively with FBS (r=0.467, p<0.0001), HbA1C (r=0.269, p=0.002), TC (r=0.513, p<0.0001), TG (r=0.530, p<0.0001), LDL (r=0.518, p<0.0001), and diabetes duration (r=0.198, p=0.002), and negatively with HDL (r=-0.288, p<0.0001). Hypertriglyceridemia and hypercholesterolemia were present in 63.07% and 36.15% of patients, respectively. NAFLD was identified in 40 patients (30.76%).

Overall, significant hyperglycemia and dyslipidemia were associated with elevated ALT (30.76%), AST (51.54%), and GGT (30.76%). These findings are consistent with Han N et al., who highlighted the role of hyperlipidemia in LFT abnormalities in T2DM [19]. Other reports confirm that dyslipidemia, particularly elevated LDL and reduced HDL, is strongly linked to T2DM and abnormal LFTs [20-22].

Fat accumulation in hepatocytes is a common diabetes-related complication, affecting 40-70% of patients. When accompanied by inflammation and steatonecrosis, it may drive persistent enzyme elevation. In our sample, AST (51.54%, 95% CI: 29-64.2%) was the most common abnormality, followed by ALT (30.76%, 95% CI: 28.5-42.63%) and ALP (56.93%, 95% CI: 45.5-65.2%). Abnormalities were more frequent in men than women, possibly due to hormonal differences. Prolonged diabetes duration also contributed to worsening liver health [23].

Logistic regression analysis confirmed independent associations of abnormal TC (OR: 1.45, 95% CI: 1.29-1.62), LDL-C (OR: 1.59, 95% CI: 0.41-0.69), and TG (OR: 1.80, 95% CI: 1.70-2.12) with diabetes. Furthermore, HDL metabolism is adversely affected in T2DM, contributing to cardiovascular risk. 

Ultrasonography identified fatty liver in 40 patients (30.76%), while 90 patients (69.23%) had no fatty infiltration. Patients with NAFLD had significantly higher ALT (107.93 ± 38.60 U/L), AST (69.73 ± 20.06 U/L), and total bilirubin (1.55 ± 0.24 mg/dL), with lower AST:ALT ratios (0.67 ± 0.08) compared to those without NAFLD, though ALP levels showed no significant difference. Similar associations between TG levels and NAFLD have been reported by Leite et al., where 78% of NAFLD patients had biopsy-proven NASH [24]. Hyperinsulinemia and insulin resistance may further exacerbate hepatic fat accumulation and inflammation through cytokine release (e.g., TNF) [25,26]. ROC curve analysis showed ALT had an AUROC of 0.86 in predicting NAFLD among T2DM patients, indicating good diagnostic accuracy.

Limitations

A significant limitation of this study is its dependence on hospital-based samples, which may not accurately represent the wider diabetic population in this area. The relatively small sample size and exclusion of confounders such as obesity, metabolic syndrome, and hepatitis C also limit generalizability.

## Conclusions

This study demonstrated a significant association between abnormal liver enzyme levels, fasting blood sugar, and dyslipidemia, with odds ratios indicating an increased risk of liver dysfunction in individuals with poor glycemic control. Regression analyses further highlighted strong correlations between blood glucose levels and liver function markers, including AST, ALT, and ALP. Since diabetes is a microvascular disease, progressive hepatic injury may occur as the disease advances. Incorporating LFTs and lipid profiles into the assessment of diabetes-related complications can therefore provide important insights into both hepatic damage and dyslipidemia in patients with T2DM. For patients with fatty liver disease, follow-up using laboratory indices such as FIB-4 scores and imaging modalities like elastography is recommended, as these can detect early fatty changes, advanced fibrosis, and other liver disorders. Routine screening for MASLD in diabetic patients with elevated liver enzymes may facilitate timely intervention and reduce the risk of cirrhosis and its complications. When LFT abnormalities are detected, additional evaluation of lipid profiles should also be advised to assess cardiovascular risk. Thus, regular monitoring of lipid levels in T2DM patients plays a critical role in preventing diabetes-related cardiovascular complications.
